# Influence of hepatic neoplasia on life expectancy in dogs

**DOI:** 10.14202/vetworld.2020.413-418

**Published:** 2020-03-04

**Authors:** I. F. Vilkovyskiy, Yu A. Vatnikov, E. V. Kulikov, E. D. Sotnikova, S. A. Yagnikov, S. B. Seleznev, E. A. Krotova, V. M. Byakhova, V. N. Grishin, V. P. Avdotin

**Affiliations:** Department of Veterinary Medicine, Peoples’ Friendship University of Russia (RUDN University), Moscow, Russia

**Keywords:** dogs, life span, liver tumor, metastatic tumor, primary tumor

## Abstract

**Background and Aim:**

The present study investigated the influence of liver tumor structure on life expectancy in dogs. Diseases of the liver comprise 5-25% of all non-communicable diseases in dogs, and primary hepatic tumors account for 0.6-1.3% of tumors. This research aimed to study the post-operative life span of animals with primary or metastatic tumors of the liver.

**Materials and Methods:**

During the study period, 7124 oncological operations were performed in our clinic. In total, 128 liver tumors were detected in live animals, while 323 were detected posthumously. Forty animals underwent surgery for various liver tumors. In dogs with primary liver tumors, the average age was 11.9 years and the average body weight was 15.5 kg, while in dogs with liver metastases, the mean age was 11.4 years and the average body weight was 24 kg.

**Results:**

The ratio of males to females among dogs with primary liver tumors was about 1:1 (ten females and nine males), while that among dogs with metastatic liver damage was clearly predominantly female (14 females and two males) because females often undergo surgery for cancerous mammary glands or ovaries.

**Conclusion:**

The size of tumors and the number of affected lobes had a significant effect on the post-operative life span. With a tumor size of <5 cm and a lesion covering less than two lobes of the liver, life expectancy was significantly longer and the prognosis was more favorable. In cases of large tumors or those affecting more than two lobes, life expectancy was significantly reduced and the prognosis was cautious to unfavorable.

## Introduction

In dogs, liver diseases constitute 5-25% of all non-communicable diseases, with primary liver tumors accounting for 0.6-1.3% of neoplasms [1-4]. Moreover, the liver is among the most common sites of hematogenous tumor metastases, regardless of whether the primary tumor is drained by the portal vein system or by other veins of the systemic circulation [5-8]. More than 30% of all tumors metastasize to the liver in a hematogenous way. When the primary tumor is drained by the portal vein, through which most transfer of tumor emboli to the liver occurs, the incidence of metastasis is 50% [9-13]. Several studies have established that there are three morphological categories of liver tumors in dogs [14-17]: (1) Massive tumors within a single lobe of the liver – this is the most well-verified category; (2) nodular or multifocal formations involving several lobes [18-20]; and (3) diffuse tumors occurring across the entire area of the liver [21-24]. If researchers gain more precise information about how the structural variability of liver tumors in dogs affects life expectancy, clinicians will be better able to make differential diagnoses of various liver pathologies and conduct necessary treatment on time [25-29].

The present study contributes to the differential diagnosis of liver lesions and metastatogenesis in dogs as well as to the differentiation of liver cancers from other diseases, including hereditary etiology [11,30,31].

The present research aimed to study the life expectancy of animals with primary and metastatic tumors of the liver.

## Materials and Methods

### Ethical approval and informed consent

The present study was performed in accordance with the Guide for Research on Animals and was approved by the Bioethics Commission SREC PFUR (№. 10 18.01.2018). Oral consent was obtained from all owners of animals.

### Study area

The materials for the study were animals that were admitted to the “Biocontrol” and “MedVet” veterinary clinics between October 2002 and July 2018.

The “Biocontrol” clinic was founded more than 40 years ago as part of N.N. Blokhin Cancer Research Centre of the Russian Academy of Medical Science.

### Data collection

During the study period, 7124 oncological operations were performed in the clinic. In total, 128 liver tumors were detected in live animals and 323 were detected posthumously. Forty animals underwent surgery for various liver tumors. In most patients, when liver tumors were detected, no treatment was performed because the animal’s condition was considered unpromising; most had developed metastases in other organs and had an unsatisfactory general condition at the time of admission. Some owners declined to conduct further diagnostic and treatment procedures. The average age of all the dogs was 8.6 years, and their average body weight was 19.4 kg. In dogs with primary liver tumors, the average age was 11.9 years and the average body weight was 15.5 kg, while in dogs with liver metastases, the mean age was 11.4 years and the average body weight was 24 kg.

### Statistical analysis

The obtained indices were processed using the Newman–Keuls method and the two-sided Student’s test in the Primer of Biostatistics program for Windows (ver. 4.3) (S.A.Glantz, McGraw-Hill; Appleton & Lange).

## Results and Discussion

The previous studies have shown that life expectancy depends significantly on the structural variability of liver tumors. In the present study, hepatocellular cancer was found in 11 dogs, which was 31.3% of all animals who had undergone surgery. In seven of these animals, the tumor affected more than two liver lobes and exceeded 5.1 cm in diameter at the time of diagnosis, which significantly complicated the process of treatment. In all cases, when the surgeons decided to conduct an operation, the animal’s condition was satisfactory. When the dogs showed obvious symptoms of hepatopathy and/or serious abnormalities in the biochemical and clinical blood tests, symptomatic and pathogenetic rehabilitation therapy was performed. In such cases, surgery was only performed once the animal’s clinical status and blood biochemical indicators had improved. In four cases, the tumor affected one or two lobes. In three of these, the neoplasm was in the left lobe, while the other one had the neoplasm in the right lobe (Table-1).

**Table-1:** Lifetime independence from the diagnosis and size of the tumor (days, P =0.005).

Primary neoplasm and metastasis in the liver (the number of lesions of the liver, the days of the study,** p*=*0.005)			

Up to 5.0 cm	More than 5.0 cm	Up to 5.0 cm	More than 5.0 cm
Hepatocellular carcinoma		Metastasis of ovarian cancer	
385	30	420	220
720	51	481	201
870	116	Metastasis of uterine cancer	
--	312	--	240
--	112	Metastasis of kidney cancer	
--	91	--	140
--	330	Metastasis of mammary gland cancer	
Cholangiocellular carcinoma		210	105
195	--	690	511
Hemangioma		740	480
1527	1121	630	--
1800	--	870	--
Fibrosarcoma		Metastasis of soft tissue sarcomas	
--	225	510	--
Mixed cancer		Metastasis of soft tissue sarcomas	
--	270	--	165

Numerical values - dog survival in days

Most dogs diagnosed with hepatocellular carcinoma underwent complex treatment combining lobectomy with local exposure to liquid nitrogen or alcohol. In two cases, lobectomy of one or two affected lobes was performed; in two more, only local effects were treated because the dogs showed advanced disease and had an unsatisfactory condition in the pre-operative period. In cases of right medial lobectomy, bleeding occurred because this lobe is near the caudal vena cava. In one case, the tumor invaded the wall of the vena cava, so a partial resection of the vessel wall was also carried out. The median post-operative survival period of animals with hepatocellular carcinoma was 234 days; that is, 50% of the dogs survived this period. At that time of this article’s submission, two dogs remained under our supervision; their post-operative life spans were 720 and 810 days, respectively. In both these cases, one lobe was affected. All animals were examined every 1.5-2 months. When new foci appeared or the previous ones were suspected to have grown, we performed either a repeat laparotomy (one case) or laparoscopy (two cases) to determine whether the therapeutic measures had been effective and to check for local destruction.

Cholangiocellular carcinoma was diagnosed in two dogs. In both, more than two lobes were affected at the time of diagnosis, but the tumor size did not exceed 5.1 cm. Partial or total resection of the lobe was performed in both cases, depending on the site of the tumor. In one of these dogs, ascites was diagnosed in the pre-operative period; in the early post-operative period, this animal died from profuse bleeding in the abdominal cavity. The second dog survived with treatment for 195 days, eventually dying from the progression of the underlying disease.

Hemangioma was found in six dogs in the present study. In five of these, the tumor affected one or two lobes, while one of them had more than two lobes affected. In two of the dogs, the tumors measured <5.0 cm, while in two more, they measured >5.1 cm. In three cases involving a single lesion, lobectomy or marginal resection of the lobe was performed, depending on tumor site. In other cases, combined lobectomy with cryodestruction or sclerotherapy was performed, necessitated in two cases by the need to minimize surgery due to concomitant disease (renal failure).

The median survival period of dogs with hemangioma was 1050 days. Three of them died from diseases unrelated to the underlying diagnosis. One died due to progression of renal failure. The rest of the animals were under observation when this article was submitted. Four months after surgery, one dog underwent laparotomy to search for picometers. Histological examination of the sites treated using sclerotherapy showed no tumor tissue. One dog was diagnosed with fibrosarcoma. At the time of diagnosis, the tumor affected five lobes, with a maximum size of 9 cm. After patient stabilization, combined partial resection of three lobes was performed and the rest of the neoplasms were treated using liquid nitrogen and 96% alcohol. After 30 days, a control laparoscopy with sclerotherapy of new foci was performed. The animal lived 225 days after surgery, dying due to the progression of the underlying disease.

Among all dogs in the present study, one had a mixed tumor, with simultaneous hepatocellular carcinoma and metastatic glandular cancer. The oral tumor was excised in another medical institution while we performed lobectomy of two parts and sclerotherapy of three remaining tumors that had a diameter <3 cm. The dog lived another 280 days, at which point the tumor had metastasized into the lungs (Table-2) and the animal was euthanized.

**Table-2:** Life expectancy, depending on the diagnosis and the number of tumor parts of the liver.

Degree of primary lesions and liver metastasis (the number of lesions of the liver, the days of the study,** p*=*0.005)			

1-2 lobes	More than 2-x lobes	1-2 lobes	More than 2-x lobes
Hepatocellular carcinoma		Metastasis of ovarian cancer	
330	30	420	220
720	51	--	201
870	116	--	481
--	312	Metastasis of uterine cancer	
--	112	240	--
--	91	Metastasis of kidney cancer	
--	385	--	140
Cholangiocellular cancer		Metastasis of breast cancer	
--	195	105	210
Hemangioma		690	--
1527	--	740	--
1800	--	630	--
1121	--	480	--
Fibrosarcoma		511	--
--	225	870	--
Mixed cancer		Metastasis of soft tissue sarcomas	
--	270	510	--
Metastasis of soft tissue sarcomas			
--	165		

Numerical values - dog survival in days

Four operations were performed to treat metastasis of ovarian cancer into the liver. In three of these cases, the tumor exceeded 5.1 cm and affected more than two lobes. One animal with a single lesion lived for 420 days after lobectomy and was under observation when this article was submitted, although metastases in the lungs were revealed. The median survival period of these animals was 198 days. The death of the other animals occurred due to the progression of the underlying disease.

Metastasis of uterine cancer into the liver was diagnosed in one dog at the time of primary cancer diagnosis. Specifically, two fractions were identified, with a maximum tumor size >5.1 cm. No clinical signs of hepatopathy were observed before the operation. Surgery to resect the primary tumor was performed through one-stage, supravaginal ovariohysterectomy. The metastatic neoplasms affected the left lobes of the liver. The dog lived for another 240 days, with tumor metastases affecting other parts of the liver and lungs during that time.

In one dog, metastasis of kidney cancer affected four lobes of the liver, with the maximum tumor size not exceeding 3.0 cm. Before the operation, the animal showed signs of liver failure that confirmed the results of biochemical blood tests. After stabilizing the condition and seeing positive change in the test results, we performed an operation that comprised lobectomy of one of the most affected lobes and sclerotherapy with cryodestruction to treat the remaining tumors. The animal’s life span following surgery was 102 days. The death occurred as a result of disease development.

Metastasis of breast cancer is the most common metastatic process [11,32-34]. In the present work, eight of the dogs that underwent surgery had liver metastasis of breast cancer. In all cases, the tumor at the time of diagnosis covered no more than two lobes. In three cases, the tumor measured more than 5.1 cm. In five dogs, the only lobectomy was performed, while in two cases, lobectomy was combined with local destruction. The median survival of dogs with liver metastasis of breast cancer was 480 days. One dog lived for 105 days and died as a result of disease development. One dog lived 210 days and was alive when this article was submitted, with a single metastasis in the lungs and multiple metastases in the liver. Two dogs lived 630 and 712 days, respectively. The death occurred from cancer progression in both cases. In one dog, the post-operative life span was 480 days.

Metastasis of soft-tissue sarcomas was recorded in two cases in the present study. In both cases, the tumor affected no more than two lobes, and the maximum tumor size did not exceed 5.1 cm. In one case, lobectomy, in combination with sclerotherapy, was used. In the other case, only liquid nitrogen was effective at one of the tumor sites. The dog with a lesion covering two lobes lived for 255 days after surgery and died from heart failure. The second dog survived for 510 days and died from disease development.

Metastasis of spleen fibrosarcoma occurred in one case. A splenectomy had been performed in another medical institution to treat the primary tumor 3 months earlier. At the time of diagnosis, there were multiple liver lesions, and the maximum tumor size was 18 cm. Lobectomy of one lobe was performed, the rest of the tumor and the stump of the lobe were exposed to liquid nitrogen. The post-operative life span was 165 days.

We established that the average survival period in animals with a tumor measuring <5.0 cm was 717 days, while that with tumors measuring more than 5.0 cm was 262 days. The average survival period in animals with one to two lesions was 722 days, while that in animals with more than two lesions was 200 days.

The size of tumors and the number of affected lobes have a significant effect on life expectancy. With a tumor size of <5 cm and a lesion covering less than two lobes of the liver, life expectancy significantly increases, and the prognosis is more favorable. When large tumors or lesions covering more than two lobes were detected, life expectancy was significantly reduced, and the prognosis was cautious to unfavorable. The survival of dogs with primary and metastatic neoplasms of the liver is statistically unreliable. Based on clinical and morphological analysis, we established that, in cases of multiple malignant primaries or metastatic liver tumors in dogs, the prognosis of treatment depends on the location and extent of tissue invasion. Hepatocellular cancer is more common in dogs aged 9–13 years and comprises 31% of all liver tumors [35-38].

Over 17 years, 7124 operations were performed in our institution. Specifically, 40 cancer operations were conducted, which comprised <1% of all procedures. At the same time, 21 operations were performed between 2015 and 2018. Clearly, in recent years, the number of operable cases has increased. Such a high number of operations is due to our strict sampling methods and clear definitions of indications and contraindications to surgical intervention. The recent increase in the number of operations was also made possible by timely preventive examinations of cancer patients with metastatic nodes in the liver at early stages, by ultrasound in animals with suspicions of hepatopathy, whatever the cause, and by observing oncological alertness, which also helped identify liver tumors in the first stages of development. Furthermore, experience with such operations allowed the procedures to be performed in cases of accidental findings during laparotomy for another purpose. The dominant symptoms of liver tumors are dyspepsia, manifested as anorexia, vomiting, and diarrhea. However, the first stages of primary or metastatic lesions are asymptomatic, which make it extremely difficult to detect them promptly [39-41].

## Conclusion

In the present study involving dogs with primary liver tumors, the average age was 11.9 years and the average body weight was 15.5 k. In dogs with liver metastases, the average age was 11.4 years and the average body weight was 24 kg. The ratio of males to females among dogs with primary liver tumors was about 1:1 (ten females and nine males), while females were clearly more represented among dogs with metastatic liver damage (14 females and two males) because females often undergo surgery to remove cancerous mammary glands or ovarian cancer.

Using a full range of diagnostic measures, including standard methods such as palpation, percussion, and the measurement of the necessary biochemical and clinical blood parameters, reveals the functional capacity of the liver, which must be determined before the volume of the operation can be planned. Sampling using bromsulfthalein in animals that have abnormal biochemical parameters allows clinicians more reliably to determine the absorptive-excretory function of the liver. If positive, this test can serve as a contraindication to liver resection as it indicates a risk of developing liver failure.

In the present study, more than 80% of all neoplasms of the liver were located in the left medial or left lateral lobes. In the case of large formations in the right lobes, the tumor invades the caudal hollow vein, common bile duct, and pancreas, so the animal’s condition is unsuitable for surgery and treatment is impractical [36,42,43]. Furthermore, priority on the left side of the liver can be caused by the anatomical feature of the liver’s blood circulation. It has been reliably proven that primary and metastatic malignant liver lesions have the most favorable prognosis in dogs with primary hepatocellular liver cancer [36,44,45]. Indeed, complete cure from a liver tumor is possible, but only if the malignancy is found by chance during preventive examination or ultrasound, or in operations performed for other indications; it is important to monitor abdominal organs and carry out periodic preventive examinations.

## Authors’ Contributions

IFV, SAY, YAV, and EVK had the original idea for the study and carried out the design. SBS, IFV, and SAY collected the samples. EVK, EDS, EAK, and VMB were responsible for data analysis, data cleaning. VNG, YAV, VPA, and EVK drafted the manuscript. The final draft manuscript was revised by all authors. All authors read and approved the final manuscript.
